# Prognostic significance of hemoglobin, albumin, lymphocyte, and platelet (HALP) score in breast cancer: a propensity score-matching study

**DOI:** 10.1186/s12935-024-03419-w

**Published:** 2024-07-02

**Authors:** Tongchao Jiang, Haishuang Sun, Shuyu Xue, Tiankai Xu, Wen Xia, Ying Wang, Ling Guo, Huanxin Lin

**Affiliations:** 1 Department of Radiation Oncology, State Key Laboratory of Oncology in South China, Guangdong Key Laboratory of Nasopharyngeal Carcinoma Diagnosis and Therapy, Collaborative Innovation Center for Cancer Medicine, Sun Yat-sen University Cancer Center, Guangzhou, 510060 Guangdong Province China; 2 Department of Medical Oncology, State Key Laboratory of Oncology in South China, Guangdong Key Laboratory of Nasopharyngeal Carcinoma Diagnosis and Therapy, Collaborative Innovation Center for Cancer Medicine, Sun Yat-sen University Cancer Center, Guangzhou, 510060 Guangdong Province China; 3 Department of Nasopharyngeal Carcinoma, State Key Laboratory of Oncology in South China, Guangdong Key Laboratory of Nasopharyngeal Carcinoma Diagnosis and Therapy, Collaborative Innovation Center for Cancer Medicine, Sun Yat-sen University Cancer Center, Guangzhou, 510060 Guangdong Province China; 4 Department of Vascular and Interventional Radiology, Department of General Surgery, Nanfang Hospital, Southern Medical University, Guangzhou, 510060 Guangdong Province China

**Keywords:** Breast cancer, HALP score, Prognostic, Biomarker, Survival

## Abstract

**Background:**

The hemoglobin-albumin-lymphocyte-platelet (HALP) score functions as a comprehensive index that assesses the systemic inflammatory response, nutritional, and immune status. This study aimed to explore the relationship between preoperative HALP score and the prognosis of BC patients and to develop predictive nomograms.

**Methods:**

Clinicopathological data were collected for BC patients who underwent mastectomy between December 2010 and April 2014 from Sun Yat-sen University Cancer Center. The optimal cutoff value for HALP was determined by maximally selected rank statistics for overall survival data. Propensity score matching (PSM) was applied to develop comparable cohorts of high-HALP group and low-HALP group. Kaplan–Meier curves and Cox regression analyses were performed to determine the impact of HALP on BC patients. Prognostic nomograms were developed based on the multivariate Cox regression method. Then, the concordance index (C-index), calibration plots, and decision curves analysis (DCA) were applied to evaluate the prognostic performance of the nomograms.

**Results:**

A total of 1,856 patients were included as the primary cohort, and 1,470 patients were matched and considered as the PSM cohort. In the primary cohort, the 5-year overall survival (OS) and progression-free survival (PFS) rates for high-HALP group (≥ 47.89) and low-HALP group (< 47.89) were 94.4% vs. 91.0% (*P* = 0.005) and 87.8% vs. 82.1% (*P* = 0.005), respectively. Similar results were observed in PSM cohort (5-year OS, 94.3% vs. 90.8%, *P* = 0.015; 5-year PFS, 87.5% vs. 83.2%, *P* = 0.036). Notably, multivariate Cox regression analysis in the PSM cohort showed that HALP could independently predict BC patient prognosis in both OS (HR: 0.596, 95%CI [0.405–0.875], *P* = 0.008) and PFS (HR: 0.707, 95%CI [0.538–0.930], *P* = 0.013). OS and PFS nomograms showed excellent predictive performance with the C-indexes of 0.783 and 0.720, respectively. The calibration plots and DCA also indicated the good predictability of the nomograms. Finally, subgroup analysis further demonstrated a favorable impact of HALP on both OS and PFS.

**Conclusion:**

Preoperative HALP score can be used as a reliable independent predictor of OS and PFS in BC patients, and the nomograms may provide a personalized treatment strategy.

**Supplementary Information:**

The online version contains supplementary material available at 10.1186/s12935-024-03419-w.

## Introduction

Breast cancer (BC) has the highest incidence and ranks as the second leading cause of cancer-related mortality among women, accounting for approximately 31% of all newly diagnosed cancer cases [1]. Currently, despite remarkable progress in effective therapeutic approaches, including surgical resection, endocrine therapy, and the combination of surgery with radiotherapy, chemotherapy, and immunotherapy, BC remains a formidable challenge due to its high morbidity, aggressiveness, and propensity for metastasis and recurrence [2, 3]. Accumulating evidence suggests that BC patients with the same molecular subtype and undergoing identical treatment may experience vastly different responses to treatment [4]. Overcoming tumor complexity and heterogeneity has also been accomplished through precision therapeutics, where individualized precision interventions have improved and maximized therapeutic effectiveness [4–6]. Consequently, there is an urgent need to identify alternative biomarkers that can enhance prognostic stratification and accurately predict treatment outcomes.

Emerging evidence indicates a strong association between systemic inflammation, malnutrition, and tumor progression. Several inflammatory markers, such as the neutrophil-to-lymphocyte ratio (NLR) and the platelet-to-lymphocyte ratio (PLR), have demonstrated prognostic value across various cancer types [7–9]. A meta-analysis on NLR and PLR revealed that elevated levels of pre-treatment NLR and PLR are associated with poorer overall survival (HR: 1.75, 95% CI: 1.52–2.00, *P* = 0.001; HR: 1.29, 95% CI: 1.10–1.50, *P* = 0.001, respectively) and disease-free survival (HR: 1.67, 95% CI: 1.50–1.87, *P* < 0.001; HR: 1.58, 95% CI: 1.33–1.88, *P* < 0.001, respectively) in patients with primary operable breast cancer [10]. Additionally, numerous connections exist between cancer and nutritional indicators, such as albumin (ALB) and prognostic nutritional index (PNI), reflecting the nutritional and immunological status of the body and thereby impacting the clinical outcomes of cancer patients [11–13]. Previous studies found that patients with higher albumin levels had a 45% lower risk of death compared to patients with lower albumin levels (HR: 0.55, 95% CI: 0.40–0.75, *P* < 0.001) [14]. A meta-analysis indicated a statistically significant improvement in overall survival and disease-free survival among patients with high PNI compared to those with low PNI (HR: 0.37, 95% CI: 0.27–0.50, *P* < 0.001; HR: 0.49, 95% CI: 0.25–0.96, *P* = 0.04, respectively) [15]. These inflammatory and nutritional indicators are widely adopted in clinical practice due to their simplicity, accessibility, cost-effective, and non-invasive characteristics. However, these indicators only revealed one aspect of patient’s overall health status while the final effect of treatment can be affected by various factors such as inflammatory conditions, nutritional status, and immune function of patients. This emphasizes the necessity for comprehensive and multifaceted approaches in assessing patients’ overall health and prognosis.

The hemoglobin, albumin, lymphocyte, and platelet score (HALP score), a combination of inflammatory, nutritional, and immune status, was first proposed in 2015 by Chen et al. for predicting survival outcomes of gastric cancer patients [16]. Subsequently, it has found application in predicting clinical outcomes across various malignancies in recent years [17–19]. The underlying mechanism may be attributed to the fact that HALP score represents a combination of variables related to both inflammatory, nutritional, and immune status, reflecting the condition of the body’s tumor immune microenvironment (TME) [20]. To our knowledge, equally extensive studies on the HALP score for predicting BC outcomes have not been performed thus far. Therefore, this study aimed to clarify the prognostic value of preoperative HALP score in patients with breast cancer.

## Materials and methods

### Patients

This retrospective study included 1,856 patients who underwent surgery between December 2010 and April 2014 at Sun Yat-sen University Cancer Center (SYSUCC, Guangzhou, China). Histopathological, clinical examinations data and follow-up data were obtained for all patients. The inclusion criteria were as follow: (1) diagnosis of breast cancer confirmed by pathological examination; (2) receipt of mastectomy or lumpectomy. The exclusion criterions included as follow: (1) presence of a combined primary tumor, (2) diagnosis of ductal carcinoma in situ, (3) relapse and de novo, (4) male breast cancer, (5) receipt of any antitumor therapy prior to surgery, (6) presence of hematologic, autoimmune, or acute/chronic inflammatory disorders, (7) incomplete laboratory data, and (8) lost to follow-up. The study was approved by the Research Ethics Committee of SYSUCC to ensure that the study complied with ethical guidelines and standards.

### Data collection and definition

The initial preoperative laboratory data, obtained within one week prior to the surgery, along with clinicopathological information, were extracted from the patients’ medical records. Eligible patients were re-staged according to the eighth edition of the American Joint Committee on Cancer-Tumors, Lymph Nodes, and Metastases (AJCC-TNM) staging system. The expression of estrogen receptor (ER) and progesterone receptor (PR) were scored using the St. Gallen criteria. Human epidermal growth factor receptor-2 (HER-2) status was assessed using immunohistochemistry or fluorescence in situ hybridization (FISH) tests, following the guidelines provided by the American Society of Clinical Oncology and College of American Pathologists. For HER-2 status determination, HER-2 negative status was characterized by IHC HER-2+/++ results, negative FISH findings, or cases where the FISH test was not conducted. HER-2 positive status was defined as immunohistochemistry staining with a score of 3 + or positive FISH/chromogenic in situ hybridization outcomes. Inflammatory and nutritional indicators were defined as follows: NLR = Neutrophil counts (10^9^/L) /Lymphocyte counts (10^9^/L), PLR = Platelet counts (10^9^/L) /Lymphocyte counts (10^9^/L), PNI = 10 x Albumin (g/dL) + 0.005 x Total lymphocyte counts (per mm3), HALP = Hemoglobin (g/L) × Albumin (g/L) × Lymphocytes (/L)/Platelets (/L) [21].

### Follow-up and endpoints

Follow-up was performed in all patients via (1) telephone interview with the patient or, if deceased, with family members, (2) outpatient examinations, and (3) review of hospital follow-up records. Overall survival (OS) was defined as the time from initial diagnosis to death from any cause or the final follow-up time. Progression-free survival (PFS) was defined as the time from diagnosis to the date of death, relapse, disease progression, or the final follow-up time.

### Propensity score matching

Propensity score matching (1:1) using nearest neighbor matching with caliper width equal to 0.02 was applied to develop comparable cohorts of patients with low-HALP value and high-HALP value. Covariates for matching included age, pathology, hypertension, diabetes, clinical stage, T stage, *N* stage, ER, PR, HER2, Ki-67, type of surgery, radiotherapy, adjuvant chemotherapy, endocrine therapy, target therapy, CEA, and CA153. Adequacy of matching was confirmed by comparing the propensity score distributions, and the standardized mean difference of the covariates [22].

### Statistical analysis

Statistical analyses were performed using SPSS 26.0 (SPSS, Inc., Chicago, IL) and R software (http://www.R-project.org; version 4.0.2). The optimal cut-off values for NLR, PLR, and HALP were determined by maximally selected rank statistics for survival data. ROC curve was used to compare the predictive performance of different indicators. The association between categorical variables was assessed using the Chi-square or Fisher’s exact test, while the comparison of continuous variables was conducted using the Mann-Whitney test. Survival curves were estimated using the Kaplan–Meier method, and differences between curves were compared using log-rank tests. Variables with *P* values ≤ 0.05 on univariate analysis were entered into a stepwise multivariate model to identify independent prognostic factors for OS and PFS. Analyses of OS and PFS were performed in the HALP subgroups with the use of univariate Cox regression model. Nomograms were formulated based on the results of multivariate cox regression analysis. The predictive performance of the models was further assessed using the consistency index (C-index), calibration curves, and decision curve analysis (DCA). Two-tailed *P* values < 0.05 were considered to indicate statistical significance.

## Results

### The optimal cutoff value of NLR, PLR and HALP value and comparison of predictive performance of different indicators

**Fig. 1:**
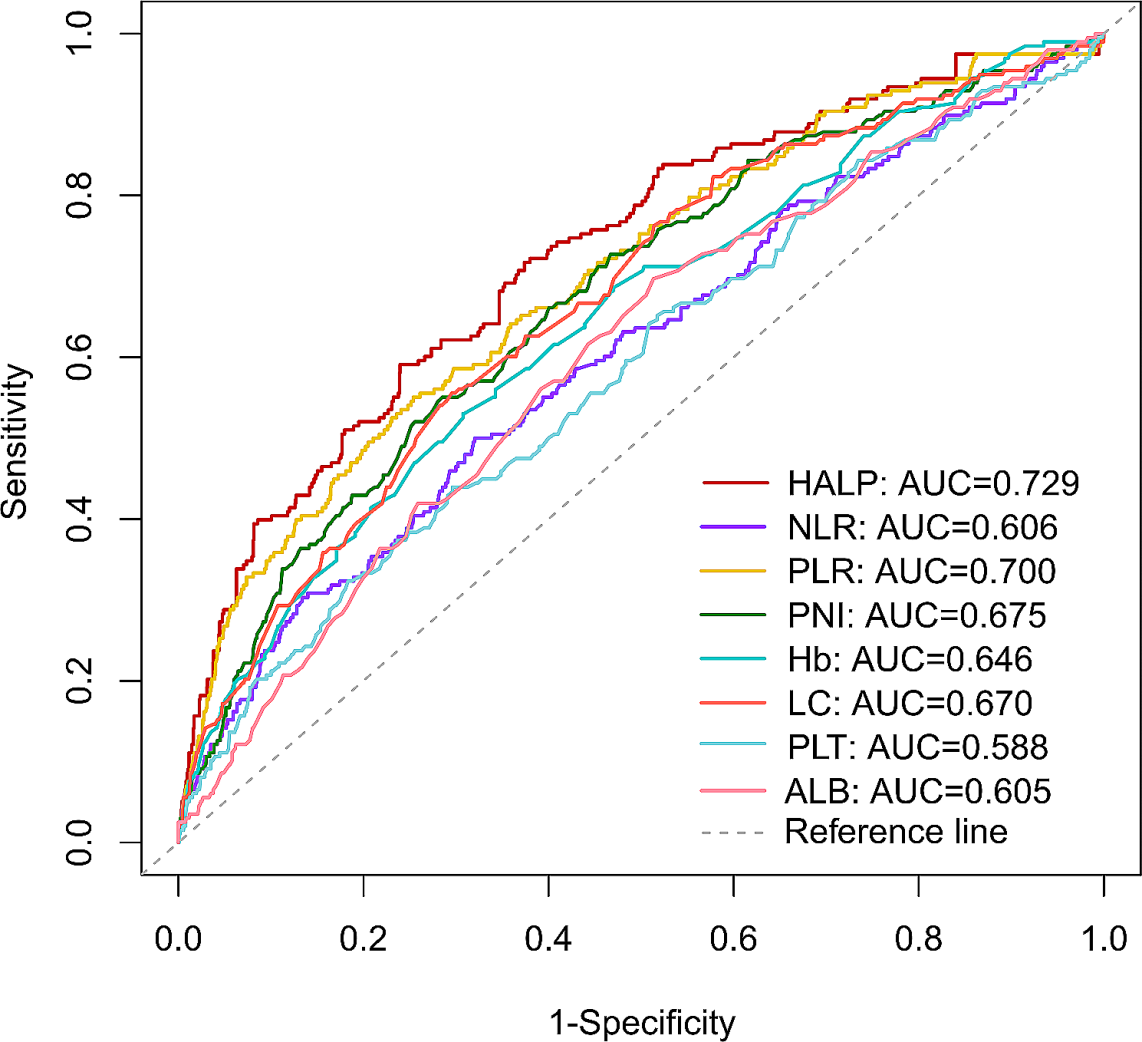
ROC curves of different inflammatory and nutritional indicators for predicting OS. ROC, receiver operating characteristic; OS, overall survival; HALP, hemoglobin, albumin, lymphocyte and platelet; NLR, neutrophil-to-lymphocyte ratio; PLR, platelet-to-lymphocyte ratio; PNI, prognostic nutritional index; Hb, hemoglobin; LC, lymphocyte; PLT, platelet; ALB, albumin

The optimal cutoff values for NLR, PLR, and HALP were 2.94, 85.14, and 47.89, respectively, in the primary cohort by using maximally selected rank statistics (Supplementary Fig. 1). Furthermore, compared to other commonly used inflammatory and nutritional indicators, the HALP score demonstrates the better predictive accuracy in predicting OS of patients with BC, with an AUC of 0.729 (95% CI, 0.690–0.768) (Fig. 1).

### Demographic characteristics

A total of 1,856 BC patients (median age, 48 years; interquartile range [IQR], 41–57 years) were identified: 426 patients (23.0%) with stage I; 961 patients (51.8%), stage II; and 469 patients (25.3%), stage III. Based on the optimal threshold, the primary cohort was divided into two groups: low-HALP group (< 47.89, *N* = 1034) and high-HALP group (≥ 47.89, *N* = 822). The characteristics of the two groups were similar, except for age (*P* < 0.001), clinical stage (*P* = 0.026), HER-2 status (*P* = 0.041), targeted therapy (*P* = 0.010), NLR (*P* < 0.001), and PLR (*P* < 0.001) (Table 1). To obtain more reliable evidence, propensity score matching (PSM) with a 1:1 ratio was conducted to compensate for selection bias and avoid potential confounding effects, ultimately including 735 BC patients in the low-HALP group and 735 BC patients in the high-HALP group (Table 1). The propensity score distribution between the two groups achieved nearly identical after PSM adjustment (Supplementary Fig. 2). Additionally, the baseline covariates were well-balanced between the two groups after PSM, with the absolute standardized mean differences below 5% for all covariates (Supplementary Fig. 3). The results after PSM were shown in Table 1, and the clinical characteristics among the two groups were well-balanced (all *P* > 0.05).

**Table 1:** Baseline clinicopathological characteristics before and after PSM

Characteristics	Total *N* = 1856(100%)	Primary cohort			PSM cohort		
		Low-HALP group *N* = 1034(55.7%)	High-HALP group *N* = 822(44.3%)	*P*-value	Low-HALP group *N* = 735(50%)	High-HALP group *N* = 735(50%)	*P*-value
**Age(years), median (IQR)**	48(41–57)	46(40–54)	50(41–58)	**< 0.001**	48(42–56)	49(41–57)	0.890
**Age** ^**#**^				**< 0.001**			0.671
< 60 years	1555(83.8%)	903(87.3%)	652(79.3%)		619(84.2)	613(83.4)	
≥ 60 years	301(16.2%)	131(12.7%)	170(20.7%)		116(15.8)	122(16.6)	
**Pathology**				0.530			1.000
Invasive ductal carcinoma	1620(87.3%)	907(87.7%)	713(86.7%)		643(87.5)	643(87.5)	
Others	236(12.7%)	127(12.3%)	109(13.3%)		92(12.5)	92(12.5)	
**Hypertension**				0.793			0.576
No	1551(83.6)	862(83.4)	689(83.8)		608(82.7)	616(83.8)	
Yes	305(16.4)	172(16.6)	133(16.2)		127(17.3)	119(16.2)	
**Diabetes**				0.184			0.162
No	1710(92.1)	945(91.4)	765(93.1)		674(91.7)	688(93.6)	
Yes	146(7.9)	89(8.6)	57(6.9)		61(8.3)	47(6.4)	
**Clinical stage** ^**#**^				**0.026**			0.875
I	426(23.0%)	215(20.8%)	211(25.7%)		167(22.7)	175(23.8)	
II	961(51.8%)	541(52.3%)	420(51.1%)		385(52.4)	382(52.0)	
III	469(25.3%)	278(26.9%)	191(23.2%)		183(24.9)	178(24.2)	
**T stage**				0.125			0.654
T1	700(37.7%)	374(36.4%)	326(39.7%)		277(37.7)	280(38.1)	
T2	995(53.6%)	558(54.0%)	437(53.2%)		402(54.7)	398(54.1)	
T3	90(4.8%)	59(5.7%)	31(3.8%)		36(4.9)	30(4.1)	
T4	71(3.8%)	43(4.2%)	28(3.4%)		20(2.7)	27(3.7)	
**N stage**				0.195			0.858
N0	965(52.0%)	517(50.0%)	448(54.5%)		377(51.3)	389(52.9)	
N1	493(26.6%)	287(27.8%)	206(25.1%)		203(27.6)	191(26.0)	
N2	227(12.2%)	136(13.2%)	91(11.1%)		88(12.0)	84(11.4)	
N3	171(9.2%)	94(9.1%)	77(9.4%)		67(9.1)	71(9.7)	
**ER status**				0.366			0.819
Negative	546(29.4%)	313(30.3%)	233(28.3%)		213(29.0)	217(29.5)	
Positive	1310(70.6%)	721(69.7%)	589(71.7%)		522(71.0)	518(70.5)	
**PR status**				0.985			0.914
Negative	695(37.4%)	387(37.4%)	308(37.5%)		273(37.1)	271(36.9)	
Positive	1161(62.6%)	647(62.6%)	514(62.5%)		462(62.9)	464(63.1)	
**HER-2 status** ^**#**^				**0.041**			0.958
Negative	1062(57.2%)	570(55.1%)	492(59.9%)		425(57.8)	426(58.0)	
Positive	794(42.8%)	464(44.9%)	330(40.1%)		310(42.2)	309(42.0)	
**Ki-67**				0.760			0.478
< 14%	641(34.5%)	354(34.2%)	287(34.9%)		266(36.2)	253(34.4)	
≥ 14%	1215(65.5%)	680(65.8%)	535(65.1%)		469(63.8)	482(65.6)	
**Type of Surgery**				0.993			0.785
Modified radical mastectomy	1522(82.0%)	837(80.9%)	685(83.3%)		603(82.0)	607(82.6)	
Others	334(18.0%)	197(19.1%)	137(16.7%)		132(18.0)	128(17.4)	
**Radiotherapy**				0.075			0.813
Yes	508(27.4%)	300(29.0%)	208(25.3%)		192(26.1)	196(26.7)	
No	1348(72.6%)	734(71.0%)	614(74.7%)		543(73.9)	539(73.3)	
**Adjuvant chemotherapy**				0.552			0.950
Yes	1439(77.5%)	807(78.0%)	632(76.0%)		574(78.1)	575(78.2)	
No	417(22.5%)	227(22.0%)	190(23.1%)		161(21.9)	160(21.8)	
**Endocrine therapy**				0.650			0.916
Yes	1030(55.5%)	569(55.0%)	461(56.1%)		410(55.8)	408(55.5)	
No	826(44.5%)	465(45.0%)	361(43.9%)		325(44.2)	327(44.5)	
**Target therapy** ^**#**^				**0.010**			0.829
Yes	144(7.8%)	95(9.2%)	49(6.0%)		47(6.4)	45(6.1)	
No	1712(92.2%)	939(90.8%)	773(94.0%)		688(93.6)	690(93.9)	
**CEA status**				0.203			0.921
Negative	1706(91.9%)	943(91.2%)	763(92.8%)		680(92.5)	679(92.4)	
Positive	150(8.1%)	91(8.8%)	59(7.2%)		55(7.5)	56(7.6)	
**CA153 status**				0.311			0.658
Negative	1658(89.3%)	917(88.7%)	741(90.1%)		667(90.7)	662(90.1)	
Positive	198(10.7%)	117(11.3%)	81(9.9%)		68(9.3)	73(9.9)	
**NLR**			**< 0.001**				**< 0.001**
Low	772(74.7%)	781(95.0%)			546(74.3%)	698(95.0%)	
High	262(25.3%)	41(5.0%)			189(25.7%)	37(5.0%)	
**PLR**			**< 0.001**				**< 0.001**
Low	0(0%)	297(36.1%)			0(0%)	246(33.5%)	
High	1034(100%)	525(63.9%)			735(100.0%)	489(66.5%)	

PSM, propensity-score matching; HALP, hemoglobin, albumin, lymphocyte, and platelet; NLR, neutrophil-to-lymphocyte ratio; PLR, platelet-to-lymphocyte ratio

### Prognostic value of HALP for OS and PFS

**Fig. 2:**
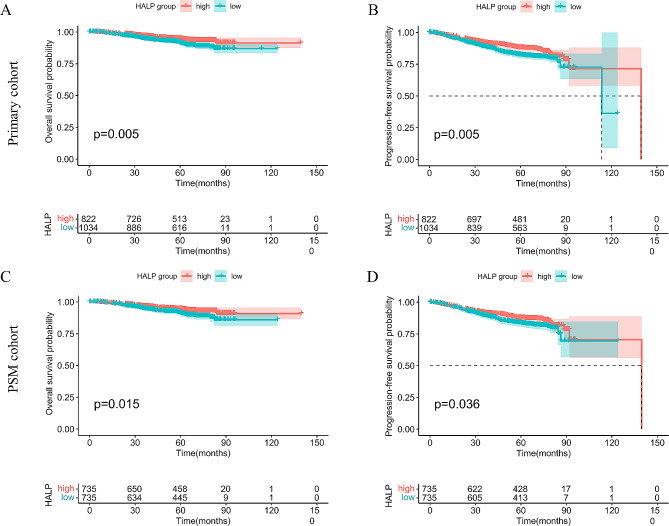
Kaplan Meier survival curves for OS and PFS in primary cohort and PSM cohort. Kaplan-Meier curves for: (**A**) OS in primary cohort; (**B**) PFS in primary cohort; (**C**) OS in PSM cohort; (**D**) PFS in PSM cohort. OS, overall survival; PFS, progression-free survival; PSM, propensity-score matching

The 5-year OS and PFS rates for the entire cohort were 92.7% and 84.7%, respectively. In the primary cohort, the high-HALP group exhibited a significant advantage over the low-HALP group in both OS (HR: 0.607, 95%CI [0.426–0.864], *P* = 0.005) and PFS (HR: 0.708, 95%CI [0.555–0.903], *P* = 0.005; Fig. 2A, B). The 5-year OS and PFS rates for the high HALP group and the low HALP group were 94.4% vs. 91.0% (*P* < 0.05) and 87.8% vs. 82.1% (*P* < 0.05), respectively. After PSM adjustment, the similar result was observed in both OS (HR: 0.623, 95%CI [0.524–0.914], *P* = 0.015) and PFS (HR: 0.748, 95%CI [0.570–0.981], *P* = 0.036) compared the high-HALP group with the low-HALP group (Fig. 2C, D). Similarly, the 5-year OS and PFS rates in the high-HALP group were longer than those in the low-HALP group (OS, 94.3% vs. 90.8%, *P* < 0.05; PFS, 87.5% vs. 83.2%, *P* < 0.05).

### Univariate and multivariate cox regression analyses

All potential predictive factors including age, pathology, hypertension, diabetes, clinical stage, ER status, PR status, HER-2 status, Ki-67, type of surgery, radiotherapy, adjuvant chemotherapy, endocrine therapy, target therapy, CEA, CA153, NLR, PLR, and HALP were included in Cox regression survival analysis. Univariate Cox regression analysis in the PSM cohort revealed that clinical stage, ER status, PR status, Ki-67, type of surgery, radiotherapy, endocrine therapy, CEA, CA153, and HALP were significantly associated with OS (*P* < 0.05; Table 2); and clinical stage, ER status, PR status, Ki-67, type of surgery, radiotherapy, CEA, CA153, and HALP were significantly associated with PFS (*P* < 0.05; Table 3). Multivariate Cox analyses results showed that HALP could independently predict patient prognosis with OS (HR: 0.596, 95% CI [0.405–0.875], *P* = 0.008; Table 2) and PFS (HR: 0.707, 95% CI [0.538–0.930], *P* = 0.013; Table 3).

**Table 2:** Univariate and multivariate analysis of OS in PSM cohort

Characteristics	Univariate analysis		Multivariate analysis	
	HR (95% CI)	*P* value	HR (95% CI)	*P* value
**Age**				
< 60	Reference			
≥ 60	1.342(0.848–2.125)	0.210		
**Pathology**				
Invasive ductal carcinoma	Reference			
Others	0.635(0.321–1.257)	0.193		
**Hypertension**				
**No**	Reference			
Yes	1.613(0.886–2.938)	0.118		
**Diabetes**				
**No**	Reference			
Yes	1.065(0.650–1.746)	0.802		
**Clinical stage**				
I	Reference		Reference	
II	2.153(1.003–4.624)	**0.049**	1.834(0.849–3.962)	0.123
III	9.211(4.418–19.203)	**< 0.001**	7.018(3.229–15.251)	**< 0.001**
**ER status**				
Negative	Reference		Reference	
Positive	0.422(0.290–0.614)	**< 0.001**	0.947(0.522–1.720)	0.859
**PR status**				
Negative	Reference		Reference	
Positive	0.353(0.241–0.517)	**< 0.001**	0.564(0.323–0.984)	**0.044**
**HER-2 status**				
Negative	Reference			
Positive	1.046(0.717–1.526)	0.817		
**Ki-67**				
< 14%	Reference		Reference	
≥ 14%	2.041(1.298–3.210)	**0.002**	1.357(0.849–2.168)	0.202
**Type of Surgery**				
Modified radical mastectomy	Reference		Reference	
Others	0.417(0.211–0.825)	**0.012**	0.715(0.356–1.43*5*)	0.345
**Radiotherapy**				
No	Reference		Reference	
Yes	1.506(1.021–2.221)	**0.039**	0.849(0.547–1.317)	0.465
**Adjuvant chemotherapy**				
No	Reference			
Yes	0.821(0.529–1.276)	0.381		
**Endocrine therapy**				
No	Reference		Reference	
Yes	0.443(0.302–0.649)	**< 0.001**	0.645(0.403–1.031)	0.067
**Target therapy**				
No	Reference			
Yes	0.913(0.424–1.964)	0.815		
**CEA status**				
Negative	Reference		Reference	
Positive	4.922(3.203–7.565)	**< 0.001**	2.547(1.587–4.087)	**< 0.001**
**CA153 status**				
Negative	Reference		Reference	
Positive	2.600(1.629–4.149)	**< 0.001**	1.239(0.746–2.057)	0.407
**NLR**				
Low	Reference			
High	1.330(0.826–2.139)	0.240		
**PLR**				
Low	Reference			
High	0.766(0.483–1.212)	0.254		
**HALP**				
Low	Reference		Reference	
High	0.623(0.425–0.914)-	**0.015**	0.596(0.405–0.875)	**0.008**

PSM, propensity-score matching; OS, overall survival; HALP, hemoglobin, albumin, lymphocyte, and platelet; NLR, neutrophil-to-lymphocyte ratio; PLR, platelet-to-lymphocyte ratio; HR, hazard ratio; CI, confidence interval

**Table 3:** Univariate and multivariate analysis of PFS in PSM cohort

Characteristics	Univariate analysis		Multivariate analysis	
	HR (95% CI)	*P* value	HR (95% CI)	*P* value
**Age**				
< 60	Reference			
≥ 60	1.208(0.856–1.704)	0.282		
**Pathology**				
Invasive ductal carcinoma	Reference			
Others	0.814(0.523–1.266)	0.360		
**Hypertension**				
**No**	Reference			
Yes	0.878(0.600-1.285)	0.502		
**Diabetes**				
No	Reference			
Yes	1.141(0.695–1.874)	0.602		
**Clinical stage**				
I	Reference		Reference	
II	1.969(1.194–3.247)	**0.008**	1.775(1.073–2.938)	**0.026**
III	7.097(4.363–11.544)	**< 0.001**	5.658(3.377–9.570)	**< 0.001**
**ER status**				
Negative	Reference		Reference	
Positive	0.637(0.481–0.844)	**0.002**	0.844(0.557–1.278)	0.423
**PR status**				
Negative	Reference		Reference	
Positive	0.580(0.443–0.760)	**< 0.001**	0.807(0.539–1.209)	0.299
**HER-2 status**				
Negative	Reference			
Positive	0.976(0.742–1.284)	0.863		
**Ki-67**				
< 14%	Reference		Reference	
≥ 14%	1.633(1.203–2.217)	**0.002**	1.301(0.950–1.782)	0.101
**Type of Surgery**				
Modified radical mastectomy	Reference		Reference	
Others	0.556(0.360–0.857)	**0.008**	0.821(0.525–1.282)	0.386
**Radiotherapy**				
No	Reference		Reference	
Yes	1.669(1.265–2.202)	**< 0.001**	0.958(0.701–1.308)	0.786
**Adjuvant chemotherapy**				
No	Reference			
Yes	1.155(0.816–1.635)	0.418		
**Endocrine therapy**				
No	Reference			
Yes	0.933(0.708–1.228)	0.620		
**Target therapy**				
No	Reference			
Yes	1.014(0.599–1.715)	0.959		
**CEA status**				
Negative	Reference		Reference	
Positive	3.703(2.630–5.212)	**< 0.001**	2.112(1.450–3.078)	**< 0.001**
**CA153 status**				
Negative	Reference		Reference	
Positive	2.392(1.684–3.399)	**< 0.001**	1.274(0.918–1.768)	0.133
**NLR**				
Low	Reference			
High	1.169(0.817–1.673)	0.393		
**PLR**				
Low	Reference			
High	0.853(0.604–1.203)	0.364		
**HALP**				
Low	Reference		Reference	
High	0.748(0.570–0.981)	**0.036**	0.707(0.538–0.930)	**0.013**

PSM, propensity-score matching; PFS, progression-free survival; HALP, hemoglobin, albumin, lymphocyte, and platelet; NLR neutrophil-to-lymphocyte ratio; PLR, platelet-to-lymphocyte ratio; HR, hazard ratio; CI, confidence interval

### Establishment and evaluation of predictive models

**Fig. 3:**
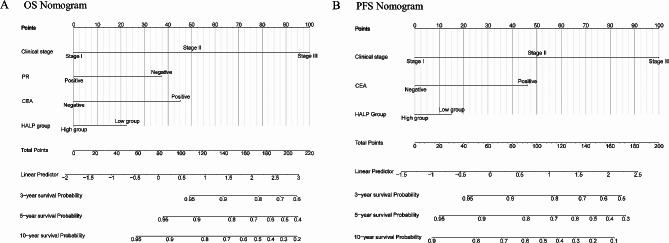
Nomograms for predicting the 3-year, 5-year, and 10-year OS and PFS of breast cancer in PSM cohort. (**A**) Nomogram for predicting OS; (**B**) Nomogram for predicting PFS. OS, overall survival; PFS, progression-free survival; PSM, propensity-score matching; HALP, hemoglobin, albumin, lymphocyte, and platelet; HALP Group, low group (< 47.89) and high group (≥ 47.89)

**Fig. 4:**
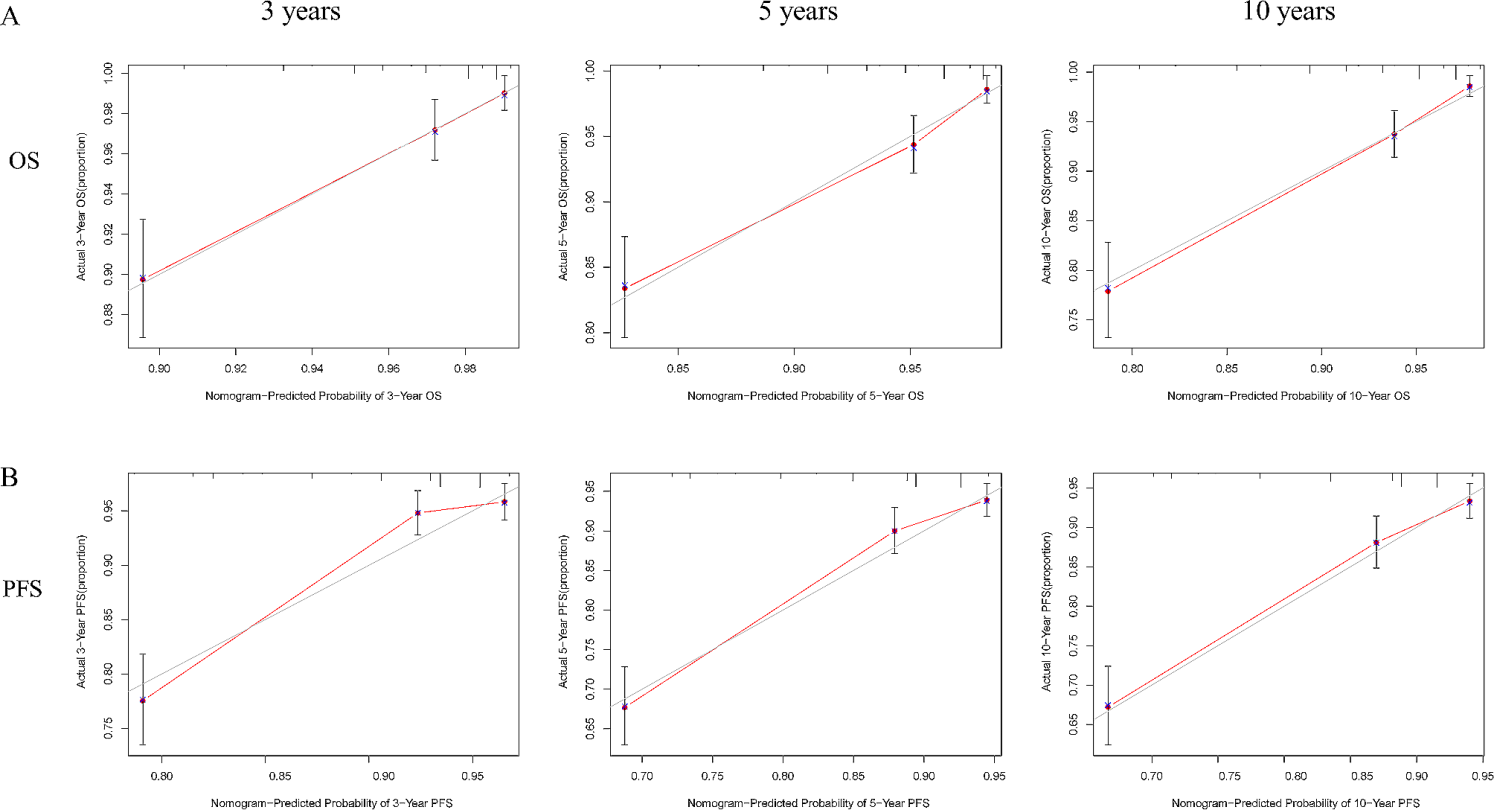
Calibration curves of OS and PFS predicting nomograms in PSM cohort. (**A**) Calibration curves for predicting 3-year, 5-year, and 10-year OS; (**B**) Calibration curves for predicting 3-year, 5-year, and 10-year PFS. OS, overall survival; PFS, progression-free survival; PSM, propensity-score matching

**Fig. 5:**
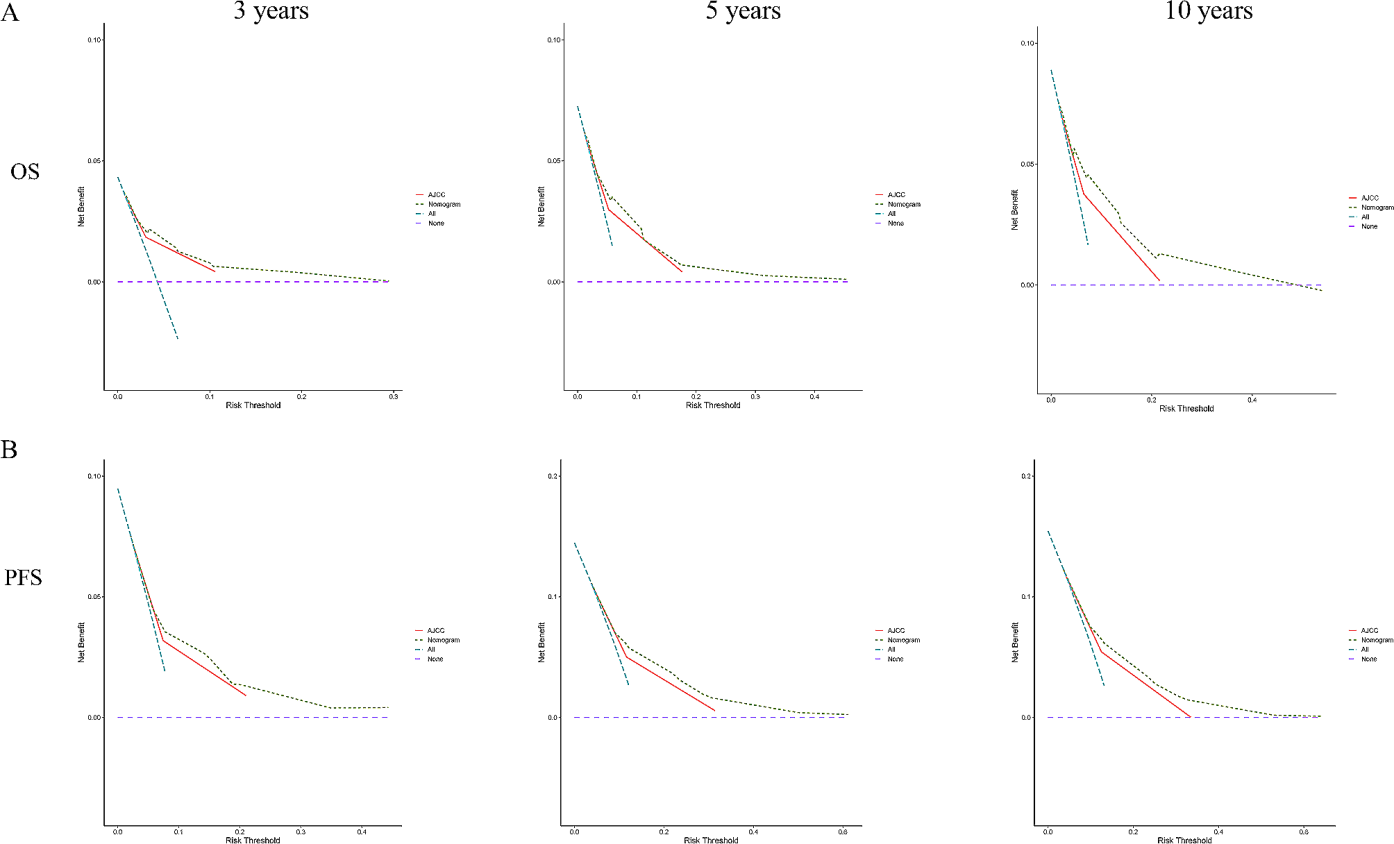
Decision curves of OS and PFS predicting nomograms in PSM cohort. (**A**) Decision curves for predicting 3-year, 5-year, and 10-year OS; (**B**) Decision curves for predicting 3-year, 5-year, and 10-year PFS. OS, overall survival; PFS, progression-free survival; PSM, propensity-score matching

Based on the abovementioned multivariate Cox regression analyses, two nomograms for the prediction of the 3-, 5-, and 10-year incidence were constructed based on four independent OS prognostic factors, including clinical stage, PR status, CEA, and HALP (Fig. 3A), as well as three independent PFS prognostic factors, including clinical stage, CEA, and HALP (Fig. 3B). The prognostic model showed excellent discrimination with C-indexes of 0.783 (95% CI [0.740–0.826]) and 0.720 (95% CI [0.684–0.756]) for predictive OS and PFS, respectively. The calibration plots of 3-, 5-, and 10-year OS and PFS demonstrated a strong consistency between predicted survival probability and actual survival proportion (Fig. 4), while the DCA curves indicated that the nomograms possess significant clinical applicability, markedly overwhelming the TNM stage (Fig. 5).

### Subgroup analysis of common clinical variables

**Fig. 6:**
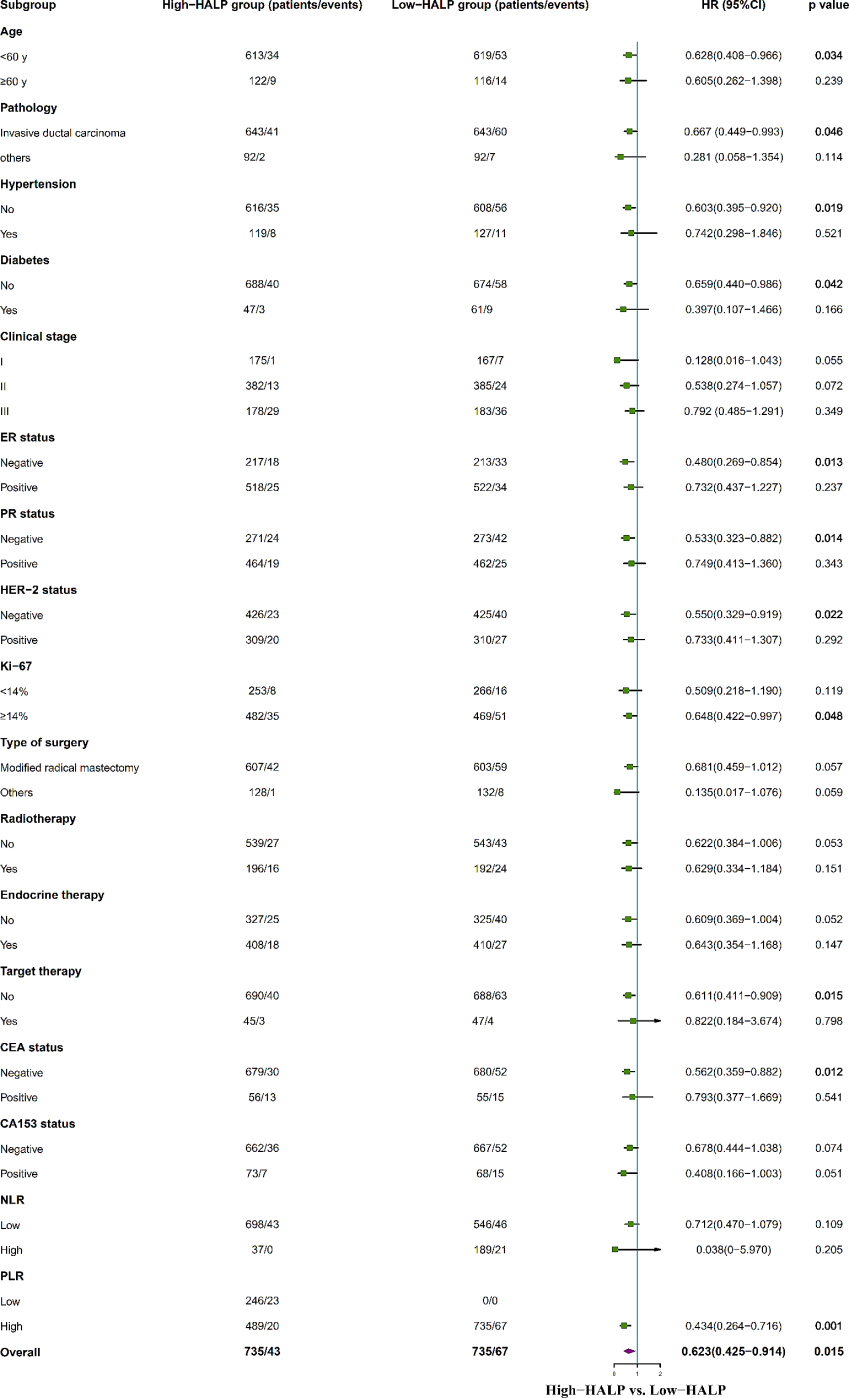
Subgroup analysis for breast cancer OS between high-HALP and low-HALP groups in PSM cohort. OS, overall survival; HALP, hemoglobin, albumin, lymphocyte, and platelet; NLR, neutrophil-to-lymphocyte ratio; PLR, platelet-to-lymphocyte ratio; PSM, propensity-score matching

**Fig. 7:**
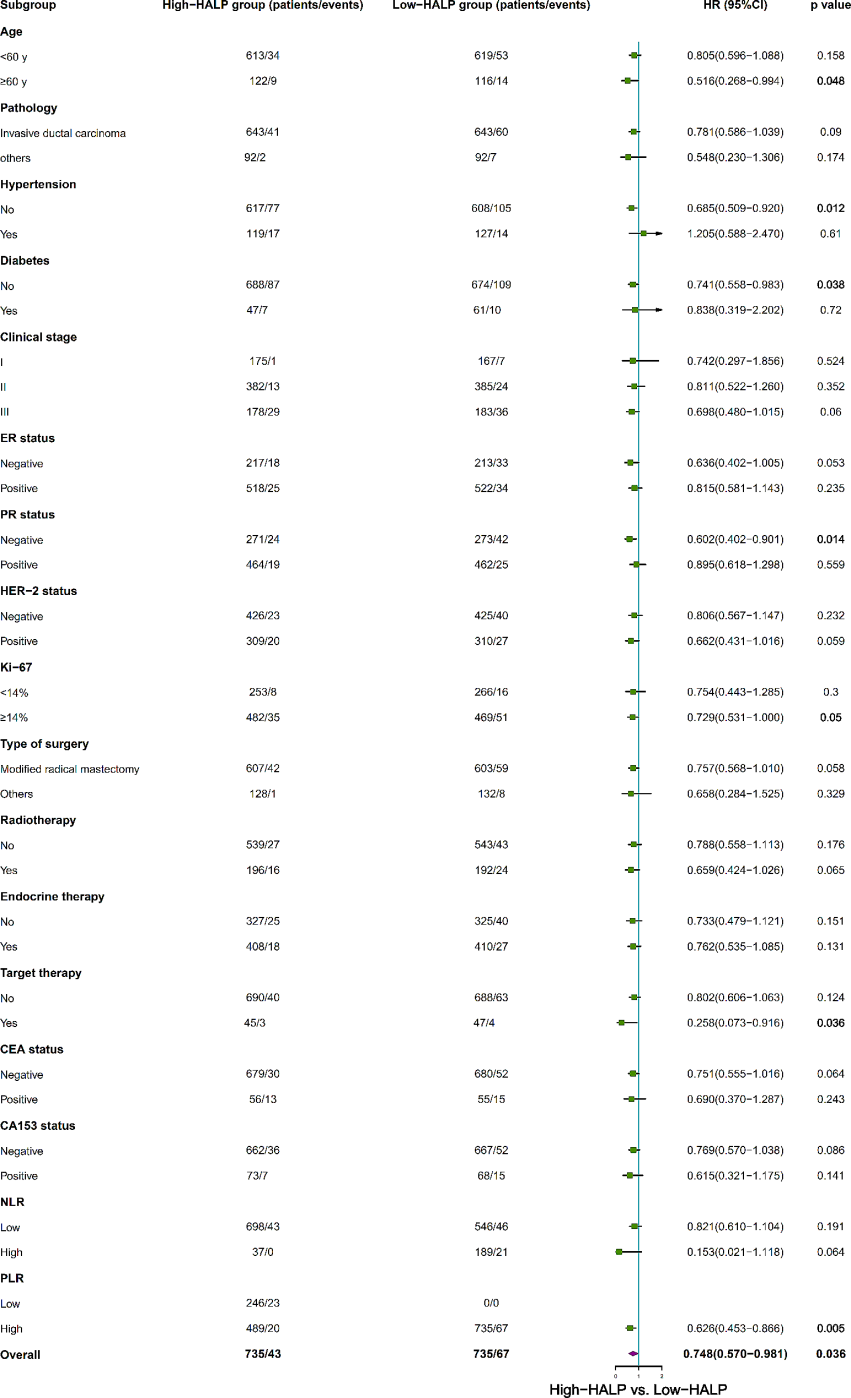
Subgroup analysis for breast cancer PFS between high-HALP and low-HALP groups in PSM cohort. PFS, progression-free survival; HALP, hemoglobin, albumin, lymphocyte, and platelet; NLR, neutrophil-to-lymphocyte ratio; PLR, platelet-to-lymphocyte ratio; PSM, propensity-score matching

Furthermore, we performed a subgroup analysis to determine if any of patient subgroups benefited from the high-HALP. In the PSM cohort, detailed subgroup analyses indicated the high-HALP group was associated with improved OS within specific subgroups, including patients under 60 years, those with invasive ductal carcinoma, those with no hypertension and diabetes, ER-negative, PR-negative, HER-2 negative, Ki-67 ≥ 14%, those not receiving targeted therapy, CEA negative, and those with high PLR, when adjustments were made for other covariates (Fig. 6). Additionally, PFS demonstrated superiority in the high-HALP group compared to the low-HALP group among patients aged over 60 years, those with no hypertension and diabetes, those with PR-negative status, individuals receiving targeted therapy, and those with high PLR (Fig. 7).

## Discussion

Identifying both prognostic and predictive biomarkers are essential for personalized oncology approaches in cancer, as these biomarkers offer insights into overall cancer outcomes, aid in cancer diagnosis, and inform treatment decisions [11, 14, 17]. In this retrospective study, we analyzed the potential clinical significance of the preoperative HALP score for breast cancer patients. First, HALP score has better predictive performance in predicting OS in BC patients than other commonly used inflammatory and nutritional indicators. Furthermore, survival analysis indicated that the high-HALP score was significantly associated with better OS and PFS both before and after PSM. Multivariable Cox regression analysis revealed the high-HALP score was an independently significant prognostic factor for both OS and PFS. Two nomograms, combined the HALP score with conventional clinicopathological factors, were established for the prediction of patients’ OS and PFS based on the results of the multivariate cox regression analyses. The performance of this models was subsequently validated with respect to discrimination, calibration curves and clinical application, demonstrating strong predictive performance for 3-year, 5-year, and 10-year OS and PFS. Additionally, subgroup analysis further demonstrated a favorable impact of HALP score on both OS and PFS.

Increasing researches indicated that inflammation is a hallmark characteristic of tumors, being intricately linked to the initiation, progression, development, and sustenance of malignancies [23–25]. Cancer-associated inflammation holds complex connections between tumors and inflammatory responses, potentially resulting in adverse prognoses and treatment response failures in cancer patients [26]. Serving as mediators of inflammatory responses, inflammatory cells play a pivotal role within the TME [27]. Malnutrition, weakening the host’s immune system functioning and cell-mediated immunity, is a prevalent condition among oncology patients, and the causes of cancer-related malnutrition are multifaceted [28]. Assessing the nutritional status of oncology patients can be challenging, particularly in clinical practice with time constraints where intricate nutritional evaluation methods may not be feasible. In recent years, exploring peripheral blood biomarkers for survival prediction has become a hot topic due to their lower cost, easy availability, and non-invasive characteristics [29], and circulating inflammatory and nutritional biomarkers such as PLR, NLR, and PNI have been significantly associated with survival and regarded as prognostic factors for various tumors [11, 28, 30].

Over the past few years, optimal predictive factors or prognostic models for breast cancer based on one or several inflammatory biomarkers have been extensively explored. However, their clinical utility remains limited due to unequal weighting of individual inflammatory biomarkers in risk scoring. In recent years, the construction of predictive models based on multiple markers instead of single inflammatory markers has garnered significant interest [18, 19, 31]. Compared to models based on one or a few inflammatory biomarkers, the integration of multiple biomarkers can enhance the predictive accuracy of prognostic models. HALP, a novel marker reflecting the inflammatory, nutritional, and immune status of the body by integrating conventional hematological markers including hemoglobin, albumin, lymphocyte, and platelets, has been employed to predict the clinical outcome of hepatocellular carcinoma [19], lung cancer [31], and esophageal cancer [17], etc. However, to our knowledge, no previous studies have reported on the prognostic significance of the HALP score in BC patients. This study demonstrates that the HALP score is an independent prognostic factor in operable BC patients, and the improvements in the HALP score are shown to significantly enhance OS and PFS in BC patients.

The intricate interplay between tumor and host immunological and inflammatory responses is closely linked to cancer-related nutrition and inflammation, and these interactions hold promise as potential targets for cancer therapies [32]. Substantial clinical evidences indicated that the low-HALP score was associated with an unfavorable prognosis [18, 19]. However, the mechanisms underlying the prognostic significance of HALP for the OS and PFS in breast cancer are not yet fully elucidated, and the physiopathologic role of hemoglobin, albumin, lymphocyte, and platelet might explain this to some extent. Hemoglobin levels serve as a crucial index for anemia. Elevated inflammatory signaling plays a significant role in the development of anemia of chronic disease [33]. Several studies have demonstrated a direct correlation between hemoglobin levels, patient survival, and tumor progression in cancer patients [33, 34]. Moreover, anemia indicated by low hemoglobin levels may lead to tumor hypoxia and treatment resistance [34]. Serum albumin, a negative acute-phase marker synthesized in the liver, serves as an indicator of nutritional status. Hypoalbuminemia can arise from multiple factors, including malnutrition, hypercatabolism, systemic inflammation, and elevated cytokine release, potentially resulting in a compromised immune response against cancer cells [35]. Numerous studies had documented an association between hypoalbuminemia and unfavorable survival outcomes across various cancer types [14, 36]. Lymphocytes play a pivotal role in the host’s anti-cancer defense mechanisms. These immune cells, capable of secreting cytokines such as interferon-γ and tumor necrosis factor-alpha (TNF-α), contribute to better prognosis by inducing apoptosis and suppressing cancer cell proliferation, invasion, and migration [32, 37]. By simultaneously activating the NF-κB and TGF-β/Smad pathways and weakening NK cell function, platelet-derived TGF-β and direct interactions between platelets and tumor cells can trigger a mesenchymal-like transition and facilitate metastasis in cancer cells [38, 39]. Collectively, these established mechanisms suggest that a higher HALP score essentially implies better nutritional status, a stronger immune response, but a weaker inflammatory response in cancer patients, ultimately resulting in a higher survival rate.

Currently, several prognostic models are utilized for assessing breast cancer patients in clinical practice, encompassing the MammaPrint Assay, PAM50 signature, and Oncotype DX [40–42]. These predictive models, to varying degrees, have limitations, such as high out-of-pocket costs and their applicability in real-world scenarios. Given the substantial economic burden and constrained accuracy, the TNM staging system continues to be the primary tool for offering treatment recommendations and conducting follow-up assessments, particularly in underprivileged regions with limited medical insurance. However, breast cancer patients with identical TNM stage classification and treatment regimens exhibited notable differences in survival outcomes owing to the remarkable heterogeneity inherent in breast tumors [2, 4]. In this study, we have developed a prognostic model that combines HALP score with other independent clinicopathological factors, achieving C-indexes of 0.783 and 0.720 for predicting OS and PFS, respectively. Compared to the previously mentioned models, our prognostic models exhibited enhanced cost-effectiveness, accuracy, and suitability for implementation in primary hospitals. Furthermore, in comparison with the widely utilized TNM staging system, our prognostic model exhibited superior predictive performance as indicated by the DCA curves. These findings collectively suggested that our predictive nomograms based on HALP score hold promise as a valuable supplement to traditional TNM staging. It may offer improved capabilities for individualized prognosis predictions and personalized clinical care guidance.

Unlike some important studies [16, 18, 19], our study performed subgroup analysis and appears to support a favorable impact of HALP on both OS and PFS in operable BC patients. A decent explanation for our observation is that patients with high-HALP score had a better performance status and anti-tumor immune response. Additionally, those in the high-HALP group may have more opportunities to receive the complete systemic treatment on time. Thus, preoperative assessment of HALP score has significant clinical value for prognosis and individualized management of BC patients.

This study still has some limitations. First, the population included in this study was from a single center, and multicenter external validation is necessary. Second, this study was a retrospective analysis, which may have been subject to selection bias and made it difficult to obtain more comprehensive clinical data, and a prospective design is particularly important for the analysis of detailed indices. Finally, we focused only on pretreatment serum indices, but failed to analyze the dynamic changes in HALP values over the entire course.

## Conclusion

In summary, we constructed a convenient and economic HALP score based on four readily available preoperative hematological and biochemical parameters, and the scores could independently predict the OS and PFS in BC patients. The prognostic nomograms utilizing the HALP score exhibited exceptional predictive accuracy and discriminative capacity, rendering them valuable as practical tools for individualized survival prognostication.

### Electronic Supplementary Material

Below is the link to the electronic supplementary material.

Supplementary Figure 1: The optimal cut-off values of NLR, PLR, and HALP for overall survival in the primary cohort by using maximally selected rank statistics

Supplementary Figure 2: Distribution of propensity score before and after propensity-score matching

Supplementary Figure 3: Standardized mean differences before and after propensity-score matching

## Data Availability

The data that support the findings of this study are available from the corresponding author.

## Abbreviations

BC: Breast cancer
HALP: Hemoglobin, Albumin, Lymphocyte, and Platelet
C-index: Concordance index
DCA: Decision curves analysis
OS: Overall survival
PFS: Progression-free survival
PSM: Propensity score matching
NLR: Neutrophil-to-lymphocyte ratio
PLR: Platelet-to-lymphocyte ratio
PNI: Prognostic nutritional index
TME: Tumor immune microenvironment
