# Supplementary figures and images for: Prognostic significance of hemoglobin, albumin, lymphocyte, and platelet (HALP) score in breast cancer: a propensity score-matching study
Source: Cancer Cell Int. 2024 Jul 2;24:230. doi: 10.1186/s12935-024-03419-w (PMC11218366; doi:10.1186/s12935-024-03419-w)

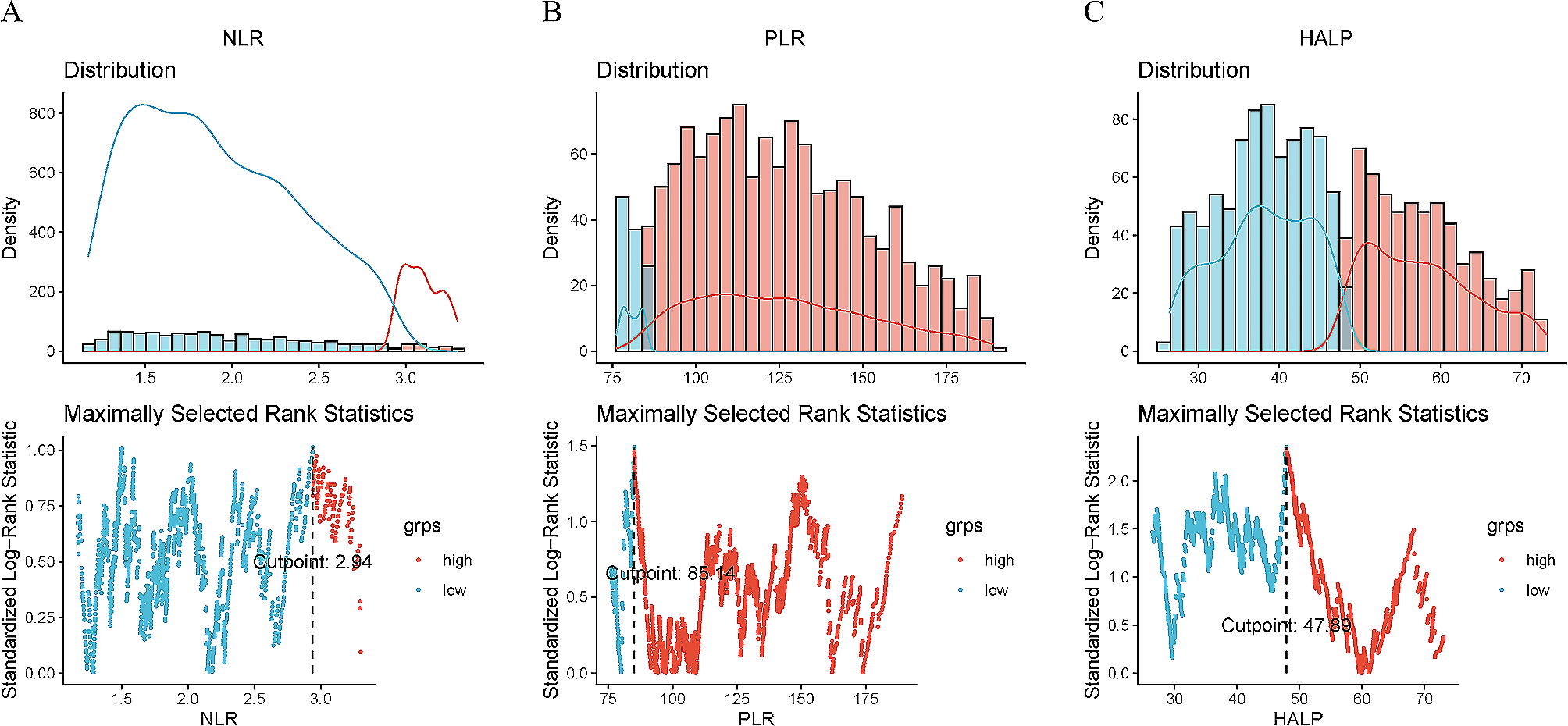

Supplement: Supplementary file 1 — Supplementary Figure 1: The optimal cut-off values of NLR, PLR, and HALP for overall survival in the primary cohort by using maximally selected rank statistics [file 12935_2024_3419_MOESM1_ESM.png]

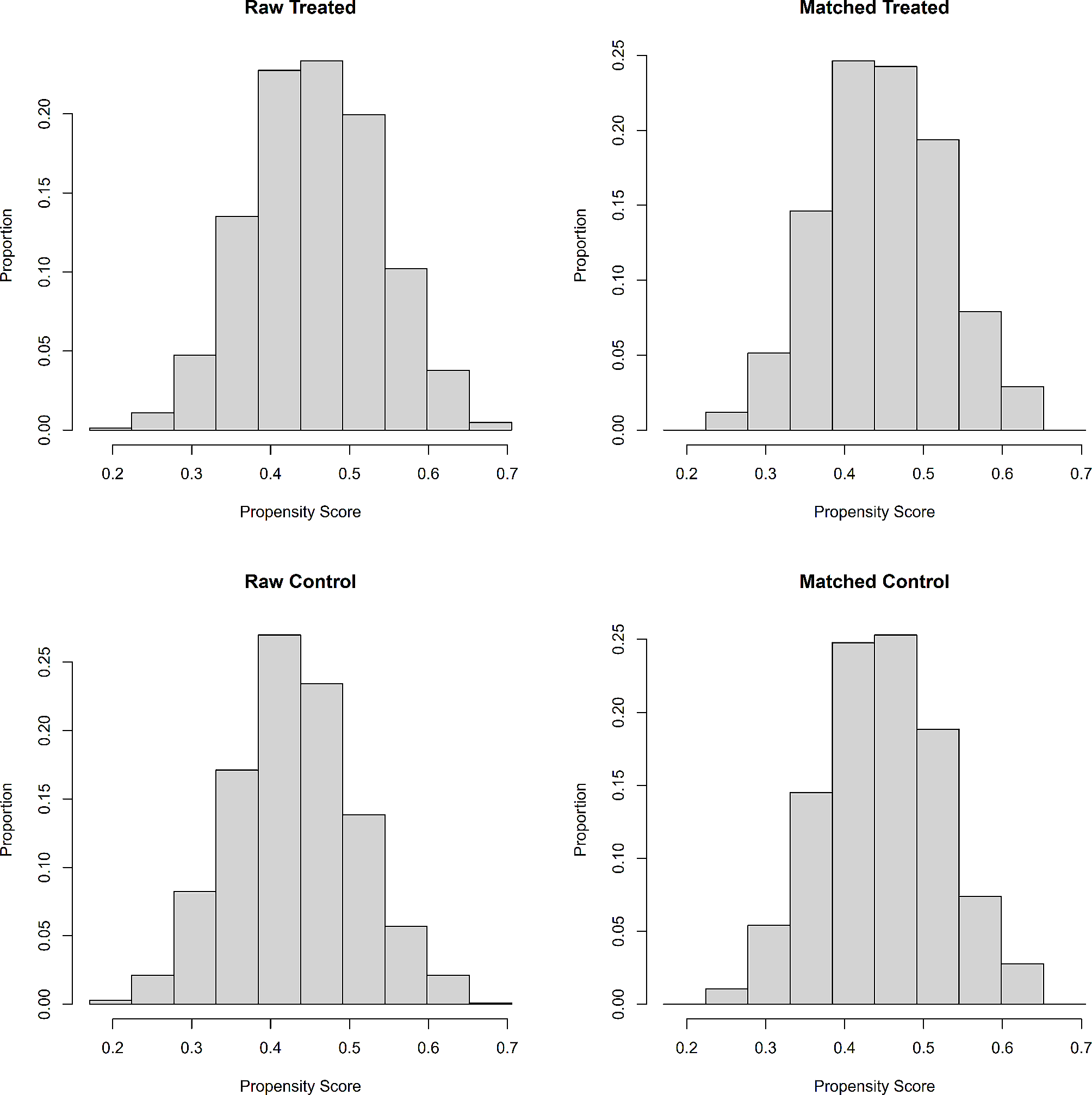

Supplement: Supplementary file 2 — Supplementary Figure 2: Distribution of propensity score before and after propensity-score matching [file 12935_2024_3419_MOESM2_ESM.png]

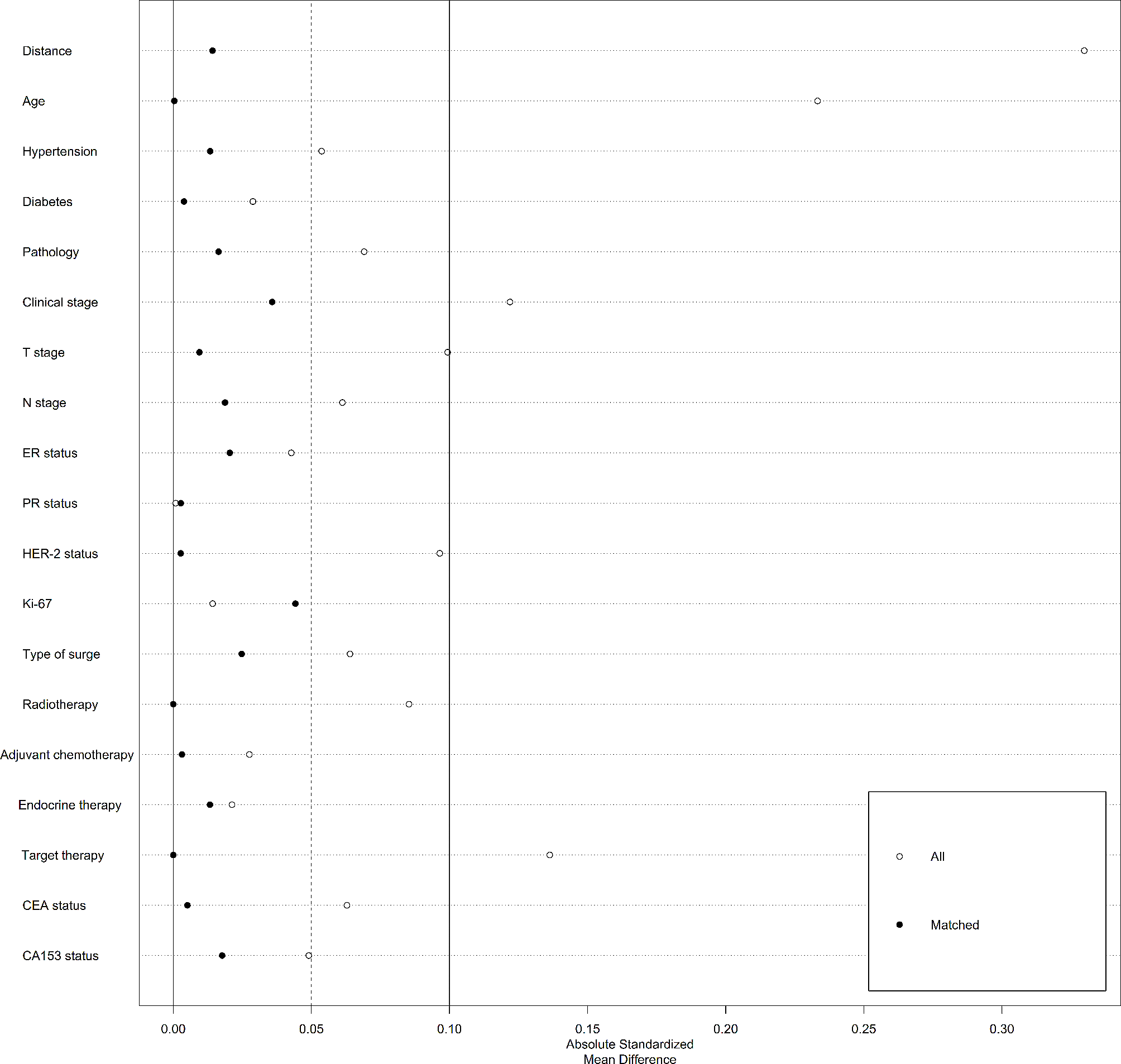

Supplement: Supplementary file 3 — Supplementary Figure 3: Standardized mean differences before and after propensity-score matching [file 12935_2024_3419_MOESM3_ESM.png]
